# Concerted Perturbation Observed in a Hub Network in Alzheimer’s Disease

**DOI:** 10.1371/journal.pone.0040498

**Published:** 2012-07-16

**Authors:** Dapeng Liang, Guangchun Han, Xuemei Feng, Jiya Sun, Yong Duan, Hongxing Lei

**Affiliations:** 1 CAS key laboratory of genome sciences and information, Beijing Institute of Genomics, Chinese Academy of Sciences, Beijing, China; 2 Graduate University, Chinese Academy of Sciences, Beijing, China; 3 UC Davis Genome Center and Department of Biomedical Engineering, University of California Davis, Davis, California, United States of America; Hospital for Sick Children, Canada

## Abstract

Alzheimer’s disease (AD) is a progressive neurodegenerative disease involving the alteration of gene expression at the whole genome level. Genome-wide transcriptional profiling of AD has been conducted by many groups on several relevant brain regions. However, identifying the most critical dys-regulated genes has been challenging. In this work, we addressed this issue by deriving critical genes from perturbed subnetworks. Using a recent microarray dataset on six brain regions, we applied a heaviest induced subgraph algorithm with a modular scoring function to reveal the significantly perturbed subnetwork in each brain region. These perturbed subnetworks were found to be significantly overlapped with each other. Furthermore, the hub genes from these perturbed subnetworks formed a connected hub network consisting of 136 genes. Comparison between AD and several related diseases demonstrated that the hub network was robustly and specifically perturbed in AD. In addition, strong correlation between the expression level of these hub genes and indicators of AD severity suggested that this hub network can partially reflect AD progression. More importantly, this hub network reflected the adaptation of neurons to the AD-specific microenvironment through a variety of adjustments, including reduction of neuronal and synaptic activities and alteration of survival signaling. Therefore, it is potentially useful for the development of biomarkers and network medicine for AD.

## Introduction

Alzheimer disease (AD) is characterized neuropathologically by the excessive accumulation of cerebral Aβ amyloid plaques and neurofibrillary tangles (NFTs). Currently, the main-stream theory regarding the disease mechanism has been the amyloid cascade hypothesis [1], [2]. Particularly, soluble oligomers of Aβ have been found to be more neurotoxic than Aβ amyloid [3], [4]. Dysfunction of certain brain regions has been manifested in a variety of cognitive and behavior symptoms [5], [6]. Postmortem expression profiling of these vulnerable brain regions has revealed important clues regarding the molecular pathogenesis of AD. To explore early pathogenesis of AD, Loring et al. conducted a microarray study on the amygdala and cingulate cortex, two brain regions affected early in AD [7]. Blalock et al. investigated the disease progression by gene expression profiling of hippocampus from subjects diagnosed with incipient, moderate, and severe AD [8]., Dunckley et al. studied the systematic effect of NFT accumulation by comparing gene expression profiles of NFT-bearing neurons in entorhinal cortex with adjacent non-NFT-bearing neurons [9]. In another study, Nunez-Iglesias et al. jointly profiled mRNA and miRNA expression in parietal lobe cortex to determine their roles and the interplay in AD [10]. In a more focused study, Williams et al. performed gene expression profiling on synaptoneurosomes from prefrontal cortex of incipient AD, facilitating the understanding of synaptically localized genes [11]. Recently, in a comprehensive transcriptome study on multiple brain regions, Liang et al. examined six anatomically and functionally distinctive brain regions of AD-afflicted individuals, including the entorhinal cortex (EC), hippocampus (HIP), middle temporal gyrus (MTG), posterior cingulate cortex (PC), superior frontal gyrus (SFG), and primary visual cortex(VCX) [5]. In addition, some other studies focused on changes of gene expression pattern in single cells, peripheral blood mononuclear cells and transgenic mice [12], [13], [14].

Despite the rich transcriptome data, unveiling disease mechanism has remained a major challenge to the AD research community. Inconsistent results have been presented due to multiple sources of problems, including small sample size, measurement error, and different statistical methods. The overlap is very low for the most significantly dys-regulated genes across multiple studies. To alleviate this problem, pathway information has been incorporated in some studies. For instance, Liu et al. constructed a network of pathways to investigate the dysfunctional crosstalk of pathways in different brain regions [15]. However, these approaches are severely hampered by the insufficient pathway knowledge currently available. Due to the fast growing knowledgebase of human interactome, network-based approaches have become more powerful and informative for the study of disease mechanism [16]. Along this line, computational methods have been proposed to detect disease-related networks. Miller et al. applied weighted gene co-expression network analysis to generate co-expression subnetworks composed of genes with high topological overlap in the hippocampus of AD patients [17]. Ray et al. built an unweighted co-expression network and then used a spectral based clustering method to identify co-expression subnetworks in the entorhinal cortex of AD patients [18]. In a more recent work, they further developed a novel method to identify genes with a topological difference among co-expression networks in four AD-relevant brain regions [19].

By integrating gene expression profiles with prior protein-protein interaction (PPI) network information, others used various heuristic algorithms and scoring functions to find subnetworks transcriptionally activated or suppressed in complex diseases. In particular, Ideker et al. pioneered a method in which a significance score for each individual gene is defined based on combining multiple differential expression p-values and a given network is searched for subnetworks with high aggregated scores by simulated annealing algorithm [20]. Other groups extended this method by developing more efficient heuristic algorithms and improved scoring functions. In one of these works, greedy search was used together with a scoring function based on the co-expression information of the edge and the differential expression of the nodes to detect perturbed subnetworks in six brain regions from a pre-built AD related PPI network [21]. Encouragingly, network-based approaches to study a number of diseases have provided insights into disease mechanism and network-based biomarkers have been shown to be superior over single gene-based and pathway-based biomarkers in both accuracy and robustness [22].

In our previous study, we presented a novel approach in which a network of functional modules and canonical pathways was constructed to study the disease progression through different stages of AD [23]. However, the methodology adopted in our previous work was inadequate to identify the most critical dys-regulated genes. In this work, we applied a different approach to further examine the network perturbation at gene level on a microarray dataset for six AD-relevant brain regions [5], including EC, HIP, MTG, PC, SFG, and VCX. Since these brain region-specific gene expression data were measured in the same laboratory under the same experimental condition, they can be directly compared with each other. We shall note that brain tissues containing dead neurons were used in most of the microarray studies on AD, making it difficult to separate the cause from the consequence. This dataset, however, included NFT-free neurons by laser-capture microdissection and was considered as a key intermediate state in our previous work. Thus, this dataset provided a unique opportunity to examine “healthy” neurons living in the AD-specific microenvironment, and to reveal how they may have survived the harsh condition and/or how they may be on their way to cell death.

Combining the gene expression data with protein interactome data, we applied a heaviest induced subgraph algorithm (Heinz) with a scoring function based on differential expression p-values fitted on a beta-uniform mixture (BUM) model to find perturbed subnetworks in each brain region (a detailed flowchart illustrating the analysis procedure is provided as **Figure S1**) [24]. Compared with the scoring functions of Ideker et al. and the extended approaches, our approach explicitly separated the signal and the noise by signal/noise decomposition implemented as a BUM model which can lead to improved signal/noise ratio. Moreover, Heinz, which is based on integer-linear programming, provides exact solutions for maximal-scoring subgraph (MSS) problem, whereas previous methods can only provide approximate solutions. Based on the perturbed subnetworks, we further identified 142 hub genes, 136 of which formed a connected hub network. Since these hub genes were extracted from the perturbed subnetworks, the randomness of the identified gene dys-regulation was significantly reduced. Hub genes tend to be essential genes and conserved across species. In addition, they have the potential to affect many other genes due to higher connectivity. Hence, hub genes have been regarded as important disease-related candidate genes [25], [26], [27]. In the following sections, extensive evidence has been provided regarding the biological relevance of this hub network. Potential application of this hub network will also be discussed.

## Results

### Gene Overlap Among the Perturbed Subnetworks in Different Brain Regions

To identify the most significantly perturbed subnetwork in each brain region, we first calculated the differential expression P-values for each gene with Linear Models for Microarray Data (LIMMA) [28]. The distribution of P-values was fitted to a beta-uniform mixture model since the distribution can be considered as a mixture of signal and noise components, where the signal component is assumed to be Beta (a,1) distributed [29]. The good fitting of the data to the BUM model was demonstrated by the high consistency between the observed P-values and the expected densities under the fitted model and further supported by a Q–Q plot of the fitted distribution versus the observed P-value distribution (**Figure S2**), indicating that the signal was well-captured by the BUM model. Next, we tested the effect of FDR (false discovery rate) selection on the number of positively scored genes. When a common relaxed FDR value 0.05 was chosen, the percentage of positively scored genes was 53.8%, 52.3%, 44.1%, 44.6%, 21.3% and 9.9% for MTG, EC, HIP, PC, SFG and VCX region, respectively. The percentage of positively scored genes roughly reflected the degree of perturbation in those six brain regions, with the most significant perturbation in MTG and least significant perturbation in VCX, consistent with observation in previous studies [5].

**Figure 1:**
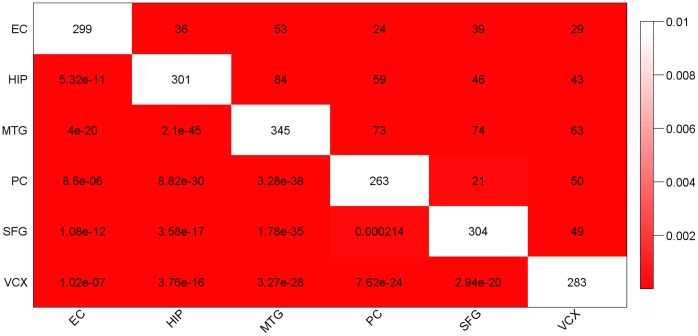
Pairwise correlation of the perturbed subnetworks in the six brain regions. The diagonal cells (white color) display the number of genes in the perturbed subnetwork of a specific brain region. Other cells of the table correspond to counts (upper right half) or significance p-values (lower left half) of overlap between a pair of brain region’s perturbed subnetworks. Coloring of the table encodes significance of overlap (p-value) by Fisher’s exact test. It is evident that all of the pairwise overlaps are significant.

Although the scale of perturbation was different in different brain regions, a shared list of perturbed genes may still exist which may reflect the most significant and reliable perturbation in AD. The extensive perturbation based on the relaxed FDR made it difficult to identify the core perturbation. To have a comparable small number of positively scored genes (∼10% of the PPI network) among the six brain regions, we decided to use different FDR cutoff for different brain regions, which was 0.00009, 0.0004, 0.0008, 0.002, 0.01 and 0.05 for MTG, EC, HIP, PC, SFG and VCX region, respectively. The relatively relaxed FDR cutoff for VCX region was consistent with the minor perturbation in this brain region [5]. Starting from these positively scored nodes (genes), Heinz algorithm was applied to search for the maximal scoring subgraph. From here on, we will refer to the maximal scoring subgraph in each brain region as the perturbed subnetwork. Based on Fisher’s test of significant gene overlap, these perturbed subnetworks identified in the six brain regions were indeed significantly overlapped with each other (**Figure 1** and **Figure S3,** an illustration of the subnetworks is shown in **Figure S4**). This overall similarity of the perturbed subnetworks in different brain regions suggests that they may constitute the core part of the dys-regulated network in AD.

### Functional Overlap Among the Perturbed Subnetworks in Different Brain Regions

**Figure 2:**
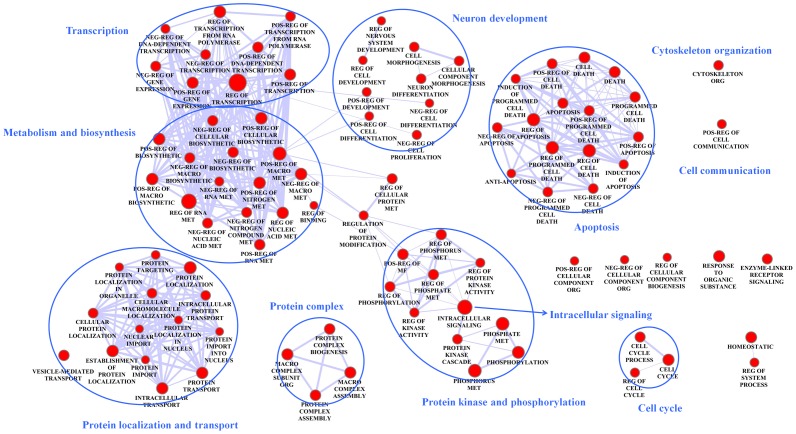
A network map of the enriched GO terms in the perturbed subnetworks. The enrichment analysis was conducted separately in each brain region’s perturbed subnetwork. Only GO terms (nodes) enriched in at least four brain regions are shown. Edge thickness represents the degree of overlap between connecting nodes (GO terms) calculated by the genes in the perturbed subnetworks. Nodes with similar functions are enclosed with red circles. Pos,Neg,Reg, Macro, met, org and MF stand for positive, negative, regulation, macromolecular, metabolism, organization and molecular function, respectively.

We carried out functional enrichment analyses for the perturbed subnetworks in the six brain regions, including Gene Ontology (GO) [30], transcription factor binding site (TFBS) and kinase substrate enrichment. Firstly, significantly enriched GO BP (biological process) terms (**Figure 2**) shared among at least four brain regions were associated with metabolism and biosynthesis, cytoskeleton organization, protein localization and transport (synaptic vesicle), transcriptional regulation, protein kinase phosphorylation, intracellular signaling, cell cycle, apoptosis, cell communication, neuron development. This is consistent with current knowledge on AD which includes elevated apoptosis, compromised cell integrity, disrupted synaptic transmission, and abnormal signal transduction and gene expression.

Secondly, we found 19 TFBS motifs enriched in at least 3 brain regions (**Table S1**), among which 16 TFBS motifs matched with known transcription factors and some have been linked to AD. Previous studies have revealed that SP1 can regulate the expression of several AD-related genes, including APP, BACE1, BACE2, and MAPT [31], [32], [33]. PAX4 variants have also been found to be associated with AD [34]. YY1 can function as transcriptional activators of BACE1 and Fe65 to facilitate the generation of Aβ via its precursor APP [35], [36], [37]. MAZ and FAC1 have been shown to co-localize to pathologic structures in AD brain. Co-expression and interaction between these two genes have biological implications for gene regulation in neurodegeneration [38]. The E2F/DP complex has been claimed to be required for Aβ-evoked neuronal cell death [39]. Loss of NRF1 in the brain can lead to dys-regulation of proteasome gene and neurodegeneration [40]. Aβ accumulation can result in suppression of activity-dependent stimulation of CREB1(CREB) and CREB/CRE-mediated gene transcription. Because CREB1 is involved in neuronal plasticity and learning, its down-regulation may contribute to the cognitive deficit in AD [41]. Both insulin resistance and oxidative stress may promote the transcriptional activity of FOXO proteins, resulting in hyperglycemia and a further increased production of reactive oxygen species (ROS) [42]. Aβ can promote neuronal apoptosis in AD by activating GSK3B, leading to degradation of β-catenin and inactivation of Wnt signaling. It has been found that lovastatin could prevent Aβ-induced apoptosis, which was accompanied by the reduction of active GSK3B, and increased nuclear translocation of β-catenin, TCF-3, and LEF-1 [43]. SOX9 plays critical role in the development of central nervous system and a regenerative treatment based on SOX9 has been proposed for AD. In addition, it has been demonstrated that SRY can exert male-specific effects in tissues other than testis, including regulating cardiovascular function and neural activity, both of which may contribute to AD development.

Thirdly, we found 18 kinases whose substrates were enriched in at least three brain regions (**Table S2**). Among these 18 kinases, CHUK and IKBKB, two components of the canonical IKK complex, are the major kinases involved in the phosphorylation of IkB proteins and crucial regulators of the canonical NF-kB pathway involved in immune response [44]. PRKCD can lead to the phosphorylation and inactivation of GSK3B and subsequent inhibition of tau phosphorylation [45]. PKMzeta, an N-terminal truncated form of PRKCZ, can accumulate in NFTs and disrupt glutamatergic synaptic transmission, leading to memory impairment in AD [46]. AKT1 can phosphorylate and inactivate GSK3B which is linked to NFT formation [47]. MAPK pathways, ERK(MAPK3) and JNK(MAPK8), are activated in AD brains and involved in the pathogenesis of AD including tau phosphorylation and amyloid deposition [48]. MARK4 is likely involved in microtubule organization in neurons and may contribute to the pathological phosphorylation of tau in AD [49]. Abnormal phosphorylation of CDK5 has also been implicated in the formation of amyloid and NFT [50]. Increased levels of RAF1 can effectively mediate Ras-dependent signals and play a critical role in the aberrant activation of the MEK/ERK pathway in AD [51]. BCR [52], [53] and CSK [54], [55], [56] variants have also been associated with AD. RIPK1, RIPK2 and RIPK3 are implicated in the regulation of apoptosis and development of AD. In particular, RIPK2 protein level is increased in the frontal cortex of AD patient and may regulate apoptosis [57]. Overall, most of these kinases are known to be associated with amyloid or tau pathology and other dysfunctions in AD.

### Identification of Hub Genes and Discovery of a Hub Network

**Figure 3:**
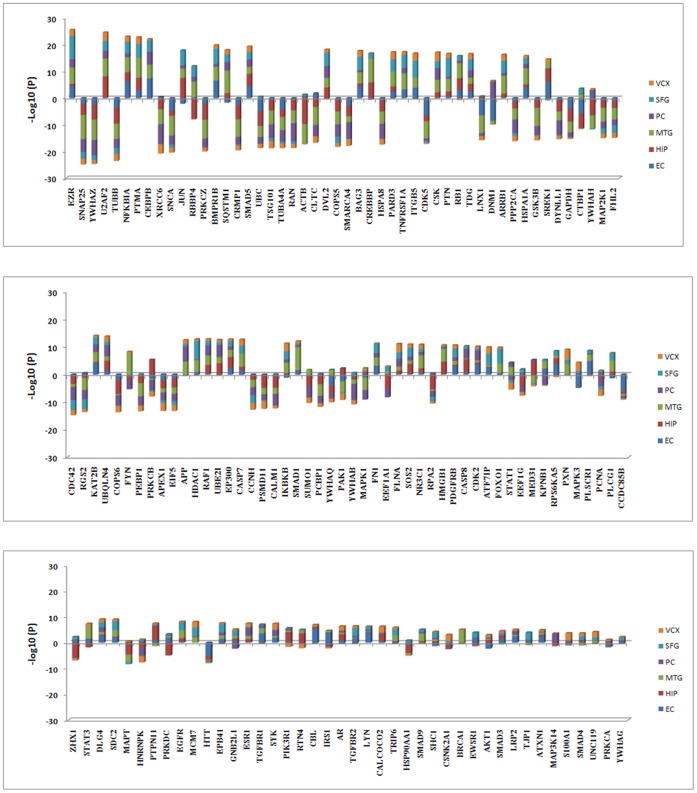
Stacked bar plots of the expression changes of the hub genes in the six brain regions. Each single colored bar represents the –log10(p-value) of a hub gene in a specific brain region (plotted above the x-axis for up-regulation, below the x-axis for down-regulation). If a hub gene is not reliably detected in certain brain regions, the significance of expression change is assigned to 0 and no color bars are displayed in the corresponding regions. The hub genes are ordered by the aggregated significance of expression change in the six brain regions.

**Figure 4:**
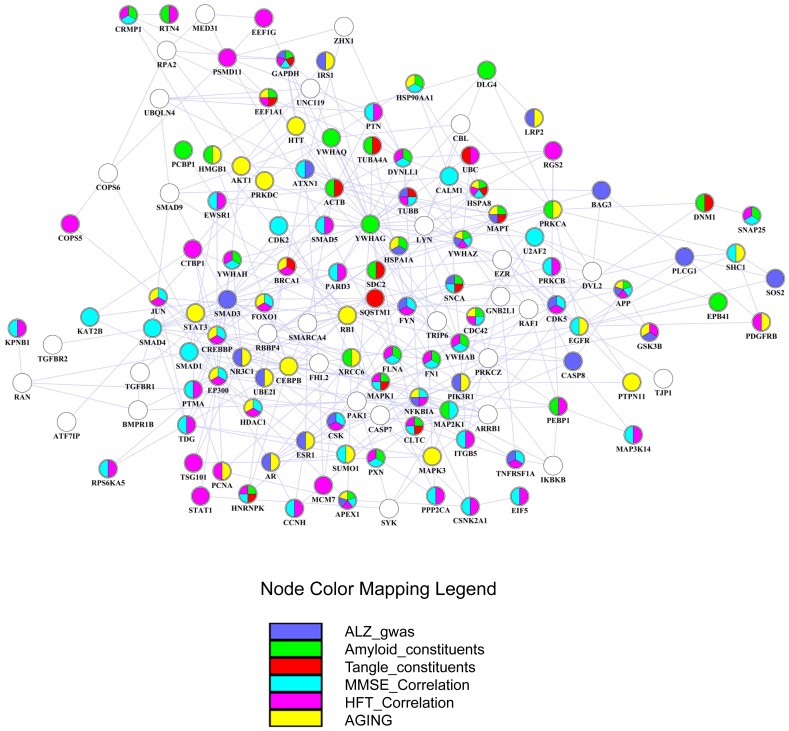
Graphical representation of the hub network consisting of 136 hub genes identified in the six brain regions. Genes are represented as nodes using various colors that represent the functional classes, including constituents of plaque or tangle, correlation with AD progression based on MMSE or NFT score, genetic risk of AD (ALZgene) and aging related genes. If a gene belongs to multiple functional classes, it will be displayed as a pie chart.

With higher connectivity, hub genes in perturbed subnetworks may play key roles in dys-regulated cellular processes. For each brain region, a gene was defined as a hub gene when the number of interactions with other genes was equal to or above the 90% quantile of the overall distribution of gene interactions in the perturbed subnetwork. By this criterion, we identified 142 hub genes from the six brain regions. To examine dys-regulation of the hub genes, we first ranked them based on their aggregated gene expression changes in the six brain regions (**Figure 3**). It was evident that most of the hub genes were perturbed in multiple brain regions. Consistent with our earlier enrichment analysis, many hub genes were functionally related to metabolism and biosynthesis, cytoskeleton component and organization, synaptic vesicle-mediated transport, transcriptional regulation, protein kinase phosphorylation, intracellular signaling, cell cycle and apoptosis. In particular, protein kinases IKBKB, AKT1, CDK5, CSK, MAPK3, PRKCZ and RAF1, whose substrates were enriched in at least three brain regions’ perturbed subnetworks, were also hub genes, suggesting that they may play vital roles in the regulation of signal transduction and tau phosphorylation in AD. Additional functional categories included heat shock protein, 14-3-3 protein family, proteins related to ubiquitin and protein degradation, learning and memory, and immune response. These hub genes potentially have an effect on AD pathology in a cooperative way, because 136 of the 142 hub genes can form a connected hub network where the pairwise interactions were present in at least one perturbed subnetwork (**Figure 4**). Many of the hub genes have been implicated in AD, and detailed interpretation of their interplay in the disease mechanism will be presented in the context of the hub network.

### Biological Relevance of the Hub Network

#### 1. The hub network is robustly and specifically perturbed in AD

We conducted analyses on several datasets to demonstrate the robust and specific perturbation of the hub network in AD (**Table S3**). First, we performed retrospective analysis on the perturbation of the hub network in the six brain regions. It was clear that the hub network was significantly perturbed in five brain regions, suggesting common disease mechanism among these regions. The insignificant perturbation of the hub network in VCX again reflected the minimal damage in this region. To validate the robust perturbation of the hub network in AD, we chose an independent dataset GSE15222 with the largest sample size so far for AD studies (364 samples) [58]. The small P-value indicated that the hub network was indeed robustly perturbed in AD.

To further test the specificity of the hub network, we collected microarray data for other neurodegenerative diseases including Parkinson’s disease (PD) and Huntington’s disease (HD), as well as a neuropsychological disorder schizophrenia (SZ) to see if the hub network is specifically perturbed in AD. Based on the same P-value cutoff (0.05), the hub network was not significantly perturbed in other related disease tissues except for the caudate region in HD. We shall note that the gene expression data from the caudate of HD was confounded by several factors, especially the significant cell loss and change of cell composition. On the other hand, the use of laser-capture microdissection technique adopted in the major dataset for AD (GSE5281) avoided these confounding factors. Overall, these analyses together demonstrated that the perturbation of the hub network was robust and in the meantime specific for AD.

#### 2. Genes constituting amyloid plaques and neurofibrillary tangles

APP and MAPT, two core genes of the hub network, were directly associated with amyloid and tau pathology. According to two independent proteomics study of laser-dissected amyloid containing plaques [59] and neurofibrillary tangles [60], 488 and 72 proteins were reported as potential constituents of plaques and tangles, respectively. From the hub network, we found 31 genes as the potential constituents of amyloid containing plaques, 13 genes as the potential constituents of neurofibrillary tangles, and 11 genes for both (**Table S4**). More literature survey revealed that additional genes such as PXN, APEX1, FN1, EPB41, HMGB1 and SQSTM1, and BRCA1 were also constituents of plaques and tangles, respectively, and SDC2 was a component of both plaques and tangles. This demonstrated that many genes in the hub network may be directly responsible for amyloid and tau pathology.

#### 3. Genes strongly correlated with AD progression

Among the widely utilized indicators of AD severity, Mini-Mental Status Examination (MMSE) score decreases with the increased severity of AD, and NFT score increases with the increased AD severity. Both scores can be used to evaluate AD progression. We tested the correlation of each hub gene’s expression with MMSE and NFT scores across all 31 subjects in a microarray study of AD progression [8]. Among the 136 hub genes, 72 genes (53%) were significantly correlated with either MMSE score or NFT score, and 43 genes (31.6%) were significantly correlated with both (**Table S5**). In addition, important transcription factors and kinases identified in the earlier enrichment analysis, including LEF1, SOX9, YY1, TCF3, TFDP1, CDK5, CSK and MAP3K3, were also correlated with either MMSE score or NFT score. This further suggested their vital roles in AD. Although the hub network was extracted from a microarray study at a specific stage of AD, our analysis indicated that it partially reflected the disease progression through different stages.

#### 4. Genes reflecting aging and genetic risks of AD

Since many genetic variations can contribute to AD development, and aging has been considered as the primary risk factor for AD, we examined the enrichment of genes related to aging and genetic risks of AD (**Table S6**). We used two curated databases: the ALZgene database [61] which provides a comprehensive, unbiased field synopsis of genetic association studies performed on AD, and the GenAge database [62], [63] which records 261 genes possibly related to human aging. We found that the hub network was enriched with AD-associated gene variants (27 out of 136, p-value 6.13e-09). Thus in addition to dys-regulation of gene expression, genetic alteration can also result in perturbation of the hub network through modification of gene product which can lead to the development of AD. In addition, we observed that the hub network was over-represented with aging-related gene (40 out of 136, p-value <2.2e-16). This suggests that strong interplay between aging and AD is reflected in this hub network which will be further discussed later.

#### 5. Potential application in the development of biomarkers and drugs

Compared with a study on the transcriptome of blood mononuclear cells by Olivier et al. (60 samples) [64], we found that hub genes STAT3, GNB2L1, SHC1, UBE2I, GAPDH, JUN, AKT1, PXN, BAG3 and DVL2 were differentially expressed in the blood of AD patients. We also re-analyzed another microarray dataset for AD with whole blood(45 samples) [65]. We found that dys-regulated hub genes including TGFBR2, NFKBIA, SOS2, HMGB1, TUBA4A, CEBPB, LYN, RAF1, HNRNPK, SNCA, TDG, TNFRSF1A and FN1. ITGB5 was also found differentially expressed in another study on the AD transcriptome of peripheral blood leukocytes [66](only a few important genes were provided in the original paper). Alternative splicing of ACTB and UBQLN4 was found in a splicing-dedicated microarray study of AD [67]. Because the expression patterns of SHC1, GAPDH, JUN, PXN, NFKBIA, HNRNPK, SNCA, TDG, TNFRSF1A, FN1 and ITGB5 were also correlated with AD progression, these genes may be potential diagnostic biomarkers in monitoring AD progression. Unfortunately these biomarkers were not reproducible in published studies, partly due to different blood cell compositions such as PBMC, whole blood and leukocytes. Further experimental validation on these potential biomarkers is undergoing in our laboratory with larger sample size and different AD severity.

Recently, it has been proposed that multi-gene drugs targeting signature networks may be more effective against complex diseases than single gene strategies [68], [69]. Our drug target enrichment analysis revealed that rapamycin and curcumin were two of the drugs with the most enriched targets in the hub network (**Table 1**). Rapamycin can rescue cognitive deficits and reduce amyloid-β Levels in AD by inhibition of mTOR signaling [70]. In the hub network, PIK3R1(PI3K subunit), AKT1, MAPK1, MAPK3, and PPPC2A (PP2A subunit) are involved in mTOR signaling. MAPK1/MAPK3(Erk1/2) and PI3K/AKT are upstream regulator of mTOR. In addition, mTOR together with insulin(IRS1)/PI3K signaling pathway can regulate PP2A and GSK3B-dependent phosphorylation of tau [71]. Targets of rapamycin also include proteins involved in progression of cell cycle, such as RB1, MCM7, PCNA, CDK2 and PCNA, and hyperactive mTOR may play a role in cell cycle re-entry in AD [72]. Curcumin has antioxidant, anti-inflammatory, and anti-protein-aggregation activities, making it an ideal candidate compound for the prevention or treatment of AD [73]. Curcumin can induce heat shock proteins and reduce protein misfolding and aggregation [74]. Target genes of curcumin also include JUN, STAT3, APP and GSK3B, suggesting a regulatory effect of curcumin in the formation of amyloid. CEBPB, a C/EBP family member, has been shown to be involved in astrocytes and microglial activation [75], [76], [77]. Translocation of STAT1 from cytosol to nucleus may also be involved in inflammatory activation in AD brains [78]. Therefore, the interaction of curcumin with CEBPB and STAT1 may have anti-inflammatory effect. Curcumin may also regulate apoptosis through its interaction with TNFRSF1A, CASP7 and CASP8. In addition, curcumin has been reported to improve learning and memory [79]. Curcumin can influence many hub genes required for memory formation and cognitive function, including CREBBP, EP300, HDAC1 and NR3C1. Curcumin targets also include steroid hormone receptor (nuclear receptor subfamily 3) AR, ESR1, NR3C1, which may protect neurons from beta-amyloid toxicity and survive a variety of coincidental insults including AD-associated neurotoxicity (detailed gene functions discussed later). In summary, our network analysis provides another layer of evidence on the potential benefits of rapamycin and curcumin on AD treatment and suggests that the hub network may be an alternative target for AD drug development.

**Table 1:** Top 5 enriched drugs for the hub network by ToppFun enrichment analysis.

Drug Name	Source	P-value	Number of genes	Target Genes
Hydrogen Peroxide	CTD	8.04E-23	38	CALM1,SMAD3,IKBKB,ATXN1,TJP1,RPS6KA5,HSPA1A,ACTB,APP,HDAC1,TNFRSF1A,RAF1,APEX1,SNCA,JUN,RB1,CASP8,FOXO1,BRCA1,MAP2K1,DYNLL1,GAPDH,HMGB1,ITGB5,FN1,PRKCZ,MAPK3,MAPK1,NFKBIA,PRKCA,PRKCB,CDK2,AKT1,SQSTM1,EGFR,STAT3,STAT1,SHC1
Rapamycin	Stitch	4.00E-21	43	TGFBR1,SMAD3,PSMD11,RPS6KA5,HSP90AA1,ACTB,PDGFRB,HTT,CBL,RAF1,FYN,MCM7,JUN,RB1,FOXO1,MAP2K1,GAPDH,FN1,PRKCZ,IRS1,MAPK3,MAPK1,NFKBIA,CCNH,PRKCA,PRKCB,BMPR1B,CDK2,AKT1,SQSTM1,EWSR1,EIF5,PIK3R1,EGFR,GSK3B,CDC42,CSNK2A1,STAT3,STAT1,PCNA,PPP2CA,NR3C1,CLTC
Curcumin	CTD	1.45E-20	38	TGFBR2,TGFBR1,SMAD3,IKBKB,SMAD4,CREBBP,APP,PDGFRB,HDAC1,CEBPB,TNFRSF1A,UBC,MCM7,JUN,RB1,CASP7,AR,CASP8,XRCC6,EP300,FOXO1,DYNLL1,FN1,PRKDC,ESR1,MAPK3,MAPK1,NFKBIA,PRKCA,AKT1,PTPN11,EGFR,GSK3B,STAT3,STAT1,PCNA,CSK,NR3C1
Doxorubicin	CTD	3.85E-20	43	MAPT,SMAD3,IKBKB,SMAD4,HSPA8,HSP90AA1,HSPA1A,CREBBP,APP,HDAC1,CEBPB,TNFRSF1A,JUN,RB1,CASP7,CASP8,FHL2,EP300,FOXO1,BRCA1,MAP2K1,TUBA4A,GAPDH,FN1,PRKDC,ESR1,HNRNPK,IRS1,MAPK3,MAPK1,TUBB,NFKBIA,PRKCA,CDK2,AKT1,SQSTM1,EGFR,GSK3B,TDG,STAT3,STAT1,PCNA,NR3C1
Resveratrol	CTD	4.46E-20	39	MAPT,IKBKB,RPS6KA5,CREBBP,ACTB,APP,PDGFRB,RAN,CEBPB,APEX1,SUMO1,SNCA,JUN,RB1,CASP7,AR,CASP8,EP300,YWHAZ,FOXO1,MAP2K1,GAPDH,ESR1,IRS1,MAPK3,MAPK1,TSG101,PLCG1,NFKBIA,PRKCA,CDK2,AKT1,PIK3R1,PTPN11,EGFR,GSK3B,CDC42,STAT3,PCNA

The P-value was evaluated by the significance of overlap between the hub genes and drug targets. The numbers of hub genes listed in the database (source) as drug targets as well as the gene symbols are provided.

## Discussion

**Figure 5:**
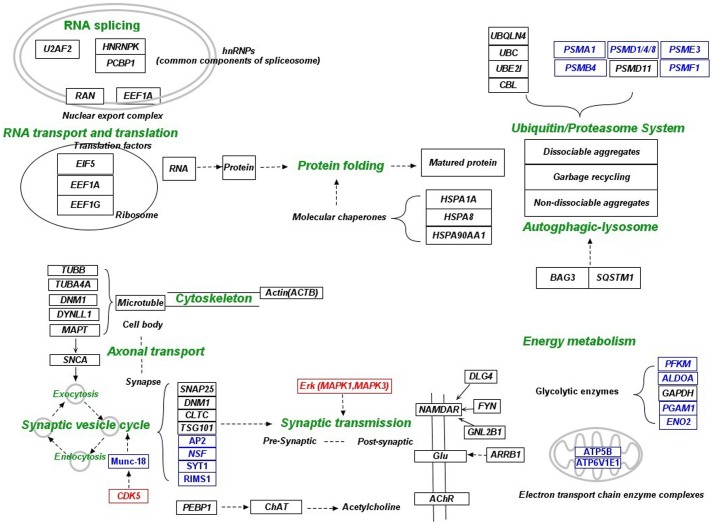
Mechanistic illustration of the adaptation strategy reflected in the hub network via manual integration of literature and KEGG pathways (part 1). The “healthy” neurons respond to the AD-specific microenvironment through the reduction of neuronal and synaptic activities. Relevant functional categories for neuronal and synaptic activities include energy metabolism, RNA splicing, RNA transport and translation, cellular recycling system, cytoskeleton (axonal transport), pre- and post- synaptic activities. Hub genes are indicated by black color. Closely related non-hub genes in the perturbed subnetwork are indicated by blue color. Transcription factors and kinases whose targets or substrates were enriched in the subnetwork are indicated with red color. Cellular functions are indicated by green color.

**Figure 6:**
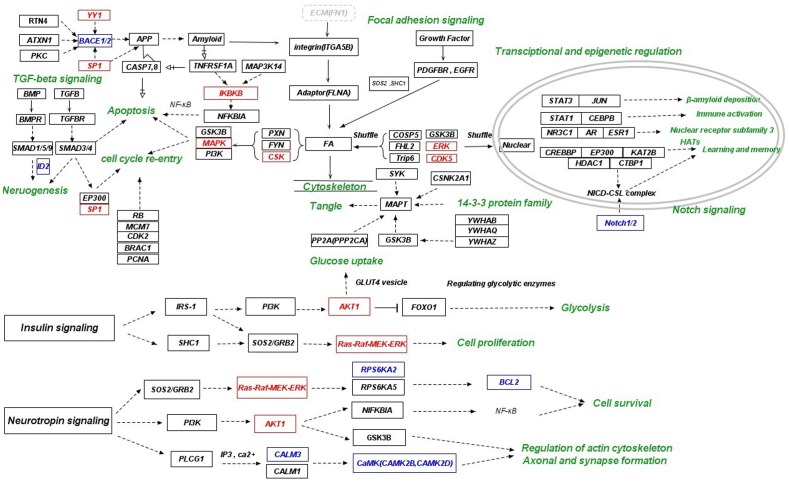
Mechanistic illustration of the adaptation strategy reflected in the hub network via manual integration of literature and KEGG pathways (part 2). The “healthy” neurons respond to the AD-specific microenvironment through alteration of survival signaling. Relevant functional categories for survival signaling include focal adhesion signaling, insulin and neurotrophin signaling, TGFB signaling, apoptosis (TNF-receptor signaling), cell cycle re-entry and genes related to amyloid and NFT formation. Hub genes are indicated by black color. Closely related non-hub genes in the perturbed subnetwork are indicated by blue color. Transcription factors and kinases whose targets or substrates were enriched in the subnetwork are indicated with red color. (other thanSP1 and YY1, these genes are also hub genes). Cellular functions are indicated by green color.

In our previous work, we proposed that the common cause of Alzheimer’s disease is likely the prolonged low supply of oxygen and nutrients in the brain [23]. In another word, Alzheimer’s disease is a complex disorder originated from energy deficiency in the brain. Brain as the most energy-demanding organ consumes about 20% of the energy of the whole human body [80]. Therefore, energy deficiency in the brain could have adverse consequence. Aging is accompanied by gradual decrease of brain perfusion starting at the age of 22. For most people, this is not sufficient to lead to AD. Rather, the aging brain can adjust to this condition by cutting down a portion of the most energy demanding activity–synaptic transmission. For some people, however, additional pathological conditions such as vascular problems will further exacerbate the situation. This will require further adjustment, trimming down more synaptic transmission, reducing intra-neuronal activities, and at the extreme case killing of certain dysfunctional neurons. For people with APOE genotype ε4, increasing evidence suggests that they have some defects in synaptic protection, including poor brain perfusion, slower recovery to anaerobic metabolism, more accumulation of amyloid, a cytoskeleton more vulnerable to damage, diminished growth and branching of neurites resulting in poor repair and worse N-methyl-D-aspartate (NMDA) excitotoxicity, all of which will render APOE ε4 carriers higher vulnerability to AD. Under this energy-centric adaptation hypothesis, we found that the hub network reflected the adaptation strategy mainly in two ways, namely reduced neuronal and synaptic activities (**Figure 5**) and altered survival signaling (**Figure 6**).

### Reduced Neuronal and Synaptic Activities

The low energy metabolism state of the neurons was manifested by the down-regulation of GAPDH, which catalyzes an important rate-limiting reaction of glycolysis, and additional non-hub genes in the perturbed subnetworks including rate-limiting enzymes of the glycolytic metabolic pathway and genes belonging to the electron transport chain. Reduction of neuronal and synaptic activities was in coordination with the low level of energy metabolism. Down-regulation of spliceosome (PCBP1 and HNRNPK), nuclear export complex (RAN and EEF1A) and translation factors (EIF5, EEF1A1 and EEF1G) suggests slowdown of RNA splicing, RNA transport and translation. Altered expression of U2AF2 may have an effect on the brain-specific splicing of APP, MAPT, UBQLN1 and BIN1 [81]. In addition, down-regulation of non-hub genes in the perturbed subnetwork related to ribosome and basal transcription were also observed (data not shown).

Many component proteins of actin and microtubule cytoskeleton, including ACTB, TUBB, TUBA4A, MAPT, DYLLN1 and DNM1, were present in the hub network. DYLLN1 is a light chain isoform of microtubule-based motor protein dynein which is involved in the impairment of axonal transport in AD [82]. Cytoskeleton provides tracks for axonal transport, thus its down-regulation may slow down the transport of lipids, proteins, mitochondria, synaptic vesicles and other cellular components such as organelles. Co-localization of MAPT and SNCA was also demonstrated in axons. It was proposed that the interaction between SNCA and MAPT could link synaptic vesicles with microtubules [83]. Down-regulation of genes related to the regulation of synaptic vesicle cycle, including hub genes CDK5, SNAP25, DNM1, CLTC and TSG101 and additional non-hub genes in the perturbed subnetwork indicated decreased pre-synaptic activities. CDK5 is involved in the regulation of synaptic vesicle exocytosis via phosphorylation of munc-18 [84]. Increased ERK activity can enhance synaptic transmission [85] and is necessary for the maintenance of learning-relevant enhancement in synaptic transmission [86]. Thus, down-regulation of CDK5 and ERK (MAPK1 and MAPK3) may reduce synaptic transmission. In addition, alteration of post-synaptic activity was shown by the dys-regulation of PEBP-1, DLG4, GNB2L1 and ARRB1. PEBP-1 can regulate choline acetyltransferase (ChAT) function which may lead to altered level of acetylcholine [87]. The significant increase of DLG4 expression may indicate a change in NMDA receptor (ionotropic glutamate receptor (iGluRs)) trafficking [88]. NMDA receptors interact with FYN through two scaffolding proteins, DLG4 and GNB2L1, which are both involved in chronic NMDA receptor hyperactivity in AD [89], [90]. ARRB1 can modulate the endocytosis of metabotropic glutamate receptors (mGluRs) and affect glutamatergic neurotransmission in AD [91]. Slow pre-synaptic anterograde transport and synaptic vesicle cycle can reduce the release of neurotransmitter, which may reduce the damage by the excessive excitation of these postsynaptic receptors.

The reduced neuronal activity was also reflected on the altered cellular recycling strategy. In conjunction with other heat shock proteins, Hsp70 (HSPA1A and HSPA8) and Hsp90 (HSP90AA1) function as chaperones to prevent protein aggregation and facilitate the proper folding of newly synthesized proteins. In addition, hub genes UBC, UBQLN4, UBE2I, CBL and PSMD11 and a few non-hub genes are recruited into the ubiquitin/proteasome system. Among these genes, polymorphism of UBE2I is associated with AD, and UBQLN4 has been found differentially expressed in AD [67] and may link ATXN1 with the chaperone and ubiquitin-proteasome pathways [92]. Inconsistent up or down-regulation of ubiquitin genes and consistent down-regulation of proteasome genes were observed. On the other hand, BAG3 and SQSTM1 were consistently up-regulated. BAG3 acts in concert with SQSTM1 to stimulate autophagy-lysosomal pathway [93]. Under acute stress conditions, when misfolded proteins accumulate and the aggregation potential increases, the ubiquitin/proteasome system might not be sufficient for the complete clearance of defective proteins. In addition, proteasome cannot degrade insoluble proteins and non-dissociable aggregates [94], and aggregates might even impair proteasome function which enhances protein aggregation [95]. Alternatively, such protein aggregates can be effectively degraded by autophagy [94]. Thus, in pathological condition of AD where increased protein misfolding and aggregation occurs, neurons may have to increasingly rely on the autophagic degradation system to maintain proteostasis. This is likely an energy-efficient way for recycling in the low energy state.

### Altered Survival Signaling

Many of the hub genes belong to focal adhesion and signaling pathways mediated by TNF receptor, TGFB, insulin and neurotrophin, all of which are involved in cell survival and death. Focal adhesion pathway was significantly activated, because ECM (FN1), integrin (ITGA5B), adaptor protein (FLNA) and FA unit (PXN, FYN and CSK) were all up-regulated. The assembly of FAK/FYN/PXN/p130cas establishes the essential four CAMs necessary for FA stabilization and integrin signaling. CSK can also be recruited to FA by FAK and PXN, because FAK and PXN contain binding sites for CSK. Fibrillar Aβ can induce integrin/FA downstream signaling that mediate cell cycle activation and cell death through different pathways involving MAPK, PI3-K and GSK3B. Signaling inputs from ECM and growth factor receptors to the FA unit in the cytoplasm can also regulate cell cycle progression. The sum of all these inputs determines the expression level of genes for cell survival and/or cell cycle progression through numerous transcriptional regulators including FOXO(FOXO1, FOXO4) and TCF(TCF3). In addition, some CAMs have been shown to shuttle between FA and nucleus to regulate gene expression, including CDK5, ERK, GSK3B and COPS5 (JAB1) and members of the LIM family of proteins (PXN and Trip6), many of which have been implicated in the molecular pathology of neurodegenerative diseases [96].

Some genes in insulin and neurotrophin signaling pathways were present in the hub network, including IRS-1, PIK3R1, AKT1, FOXO1, SHC1, SOS2, RAF1, MAP2K1, MAPK1, MAPK3, RPS6KA5, NFKBIA, GSK3B, CALM1, and PLCG1, which are involved in glycolysis, cell survival, regulation of cytoskeleton, and formation of axon and synapse. Genetic variants of nerve growth factor (NGFB), brain-derived neurotrophic factor (BDNF) and their receptors have been linked to AD [97]. Genetic variants of insulin signaling genes (including SOS2) have also been associated with AD [34]. In this work, several dys-regulated downstream genes in insulin and neurotrophin signaling pathways including IRS-1, PIK3R1, SOS2, GRB2, RPS6KA2, BCL2, NFKBIA, GSK3B and PLCG1 were also genetically associated with AD. In addition, up-regulation of genes in TGFB signaling pathway was observed, including BMPR1B, SMAD1, SMAD5, SMAD9, TGFBR1, TGFBR2, SP1 and EP300, which are involved in neurogenesis, apoptosis and cell cycle re-entry. Up-regulation of genes directly involved in apoptosis was also observed. In particular, TNF- receptor may be a crucial contributor to cell death, since a principal caspase pathway from CASP8 to CASP7 can directly contribute to neuron loss in AD brain [98], and CASP7/8 can also increase Aβ production via direct cleavage of APP [99]. Consequently, Aβ can trigger long-term death signals via CASP7/8. In addition, increased expression of MAP3K14 and TNFRSF1A can lead to activation of the IKK complex (IKBKB), which is needed to phosphorylate IκB proteins (NFKBIA) to mark them for the ubiquitination pathway [100]. Since IκB proteins inhibit NF-κB, NF-κB will thus have enhanced activation and may have downstream effects on inflammation, cell proliferation, and apoptosis [101], [102]. Several genes related to cell cycle re-entry were also dys-regulated, including RB1, MCM7, CDK2, BRAC1 and PCNA, which may provide an alternative pathway for neuronal cell death.

Recent data suggest that specific HATs and HDACs are required for memory formation [103]. CREBBP, EP300(p300/CBP) and KAT2B(p300/CBP-associated factor, PCAF) can together function as a histone acetyltransferase (HAT) to promote transcriptional regulation, and defects in their HAT activity appeared to cause problems in long-term memory formation [104], [105], [106]. PCAF homozygous KO (knock out) mice displayed short-term memory impairment at adolescent age (2 months) and gradually increasing deficits in long-term memory at later stage (6 to 12 months) [107]. Moreover, learning-induced up-regulation of CBP, p300, and PCAF has recently been associated with elevated H2B and H4 acetylation during spatial-memory consolidation [108]. In addition, HDAC1 activity seems to be neuroprotective [109]. The Notch (Notch1/2) intracellular domain (NICD) translocates to the nucleus, where it forms a complex with the DNA binding protein CSL, displacing a histone deacetylase (HDAC)-co-repressor (CoR) complex (HDAC1 and CTBP1) from CSL. HATs (CREBBP, EP300 and KAT2B), components of an activation complex, are also recruited to the NICD-CSL complex, leading to the transcriptional activation of Notch target genes which are important for learning and memory. Overall, up-regulation of HATs and HDAC1 may play a protective role in the consolidation of memory. Interestingly, NR3C1, AR and ESR1, members of steroid hormone receptors (nuclear receptor subfamily 3), are associated with aging and genetic risk of AD. Both estrogen and androgens can protect neurons from beta-amyloid toxicity via steroid receptor activation (ESR1, AR) [110]. A high density of glucocorticoid receptors (NR3C1) are contained in hippocampus which is important for memory [111], [112]. Glucocorticoids are known to influence cognitive functions and high concentrations of these steroid hormones can reduce neuron’s ability to survive a variety of coincidental insults, including AD-associated neurotoxicity [113], [114]. Thus, genetic variation of these three genes may contribute to the development of AD by affecting their protective roles.

### Brain Region Specific Perturbation

As stated earlier, the degree of perturbation in gene expression was different in the six brain regions. Overall, the most significant perturbation of the hub network was observed in MTG region (**Table S7A**). HIP region displayed similar perturbation in several functional categories, including the down-regulation of synaptic vesicle cycle, cytoskeleton, NFT related genes and RNA transport and translation. The dys-regulation of insulin and neurotrophin signaling and cellular recycling system also displayed high consistency in these two brain regions. The major difference in these two regions was the significant up-regulation of focal adhesion and TGFB signaling only in MTG region. This suggests that these two brain regions adopt similar response mechanism while higher level of stress likely exists in MTG region. The aging related SFG region did not show significant down-regulation in neuronal and synaptic activities. However, it still displayed certain level of dys-regulation in the survival signaling pathways, suggesting the existence of pathological condition in SFG region. VCX region only displayed significant dys-regulation in synaptic vesicle cycle, focal adhesion and cellular recycling system, suggesting marginal pathological condition in this region. In addition, energy metabolism related gene GAPDH was mainly down-regulated in MTG, HIP and EC regions, consistent with the more severe pathological condition in these three regions.

### The Interplay between Aging and AD

In order to understand the interplay between aging and AD, we examined the dys-regulation of the hub network in an aging related microarray dataset GSE11882 (**Table S7. A**) [115]. Although gene expression in four brain regions was measured, the dys-regulation of the hub network was mainly restricted to SFG region during aging (slightly less perturbation in PCG region). Interestingly, for most of the hub genes, the direction of the dys-regulation was consistent in aging and AD, including down-regulation of synaptic vesicle cycle, cytoskeleton, RNA transport and translation and NFT related hub genes, up-regulation of focal adhesion, TGFB signaling, HATs learning and memory, TNFR signaling and cell cycle re-entry, as well as dys-regulation of insulin and neurotrophin signaling. This suggests that the most significant change of microenvironment is in SFG region during aging, which is switched to MTG region during the development of AD. The similar neuronal response suggests similarity exists in the change of microenvironment, and in some sense, AD can be regarded as aging spreading to more brain regions. Nevertheless, notable difference was observed, including the up-regulation of genes involved in FA and nucleus (FA/N) shuttling and down-regulation of APP related genes. This suggests that FA/N shuttling genes are actively involved in neuronal response during aging, and the low production of APP is part of the response mechanism. In addition, consistent up-regulation in three brain regions (SFG, HIP and PCG) was observed for FLNA, PXN, BAG3, TNFRSF1A, CASP7, BMPR1B, SMAD9, CDK2 and NFKBIA, suggesting a concerted effort among these signaling genes on coping with aging-induced change of microenvironment in multiple brain regions. Although some energy metabolism related genes were not significantly dys-regulated during aging, the observed similar adaptation is still likely a response to the deficiency of energy sources. This could be a common problem for the whole brain during aging. However, the deficiency level may be less severe in other brain regions so that systematic adjustment can be avoided. In AD, somehow other pathological conditions make the deficiency problem more prominent in MTG, HIP and a few other brain regions, which triggers systematic response in those brain regions.

### Perturbation Related to the Formation of Amyloid and Tangle

In a recent experimental work, it was found that intra-neuronal Aβ level increased in Braak stages I to III but decreased significantly in later stages [116]. In this work, we also found an intriguing pattern of APP expression level (**Table S7. A**). APP expression was significantly down-regulated in SFG region during aging, up-regulated in MTG region at the “intermediate” stage, and again down-regulated at the late stage. It’s likely that APP expression level is a critical component of the adaptation strategy. It has been proposed that Aβ can be protective due to its role in the enhancement of synaptic transmission. However, high concentration of Aβ can trigger the formation of toxic oligomers and amyloid. Therefore, the purpose of higher APP level at the “intermediate” stage may be to enhance synaptic transmission, and the purpose of lower APP level at late stage may be to avoid excessive amyloid accumulation. Nevertheless, exactly how APP expression is regulated by SP1 and other transcription factors and how it’s connected to focal adhesion and other signaling pathways can not be easily deciphered from the microarray data. Beyond APP expression, RTN4 [117], ATXN1 [118] and PKC [119], [120], which can regulate the proteolytic processing of the APP by BACE (**Figure 5**), were also found dys-regulated, adding another layer of complexity to the understanding amyloid formation. STAT3 can bind to the promoter region of BACE and increase the expression of BACE, leading to higher production of Aβ [121]. In addition, differential expression and phosphorylation of JUN has been observed in AD [122], [123], and JUN can participate in the cascade of events leading to increased APP and β-amyloid deposition in AD [124].

14-3-3 proteins (YWHAB, YWHAH, YWHAQ, and YWHAZ) have been found in NFT [125]. Among them, YWHAZ is the most extensively studied, its polymorphism is associated with AD [126], [127], and it can stimulate tau phosphorylation by GSK3B [128]. In addition, YWHAB is associated with the development of the 3-repeat NFT in AD [129], and YWHAQ can mediate tau phosphorylation by SGK1 [130]. 14-3-3 proteins were significantly down-regulated at the “intermediate” and late stages. Insignificant perturbation of 14-3-3 protein in SFG and VCX regions may partially explain why VCX is pathologically spared. The decreased level of CSNK2A1 has been reported to be associated with the aberrations in tau phosphorylation [131]. Unique up-regulation of CSNK2A1 in the VCX region may be part of the resistance mechanism against tangle formation. Another hub gene Syk can also phosphorylate tau [132]. Moreover, an important tau kinase GSK3B was significantly down-regulated at the “intermediate” stage but up-regulated at the late stage, suggesting its critical role in NFT formation. To further examine the events related to NFT formation, we analyzed gene expression in two additional microarray datasets where tangle-bearing and tangle-free neurons were compared (**Table S7. B**) [9], [133]. We observed significant dys-regulation of seven hub genes in one dataset and two more in the other dataset, among which two were present in the insulin signaling pathway, including the up-regulation of IRS1 and FOXO1. This suggests that the energy sensing pathway is intimately connected to the accumulation of NFT [71]. This could be a self-killing strategy when neurons sense a highly stressful microenvironment. The down-regulation of PPP2CA which can dephosphorylate tau may also be part of this self-killing strategy. In addition, the dys-regulation of RAF1 and NFKBIA suggests the involvement of neurotrophin signaling pathway in tau regulation.

### Critical Genes in AD Pathogenesis

Based on the above analyses of the hub network, we selected a few genes that may play more important roles in AD pathogenesis (**Table S7. C**). Since the reduction of neuronal and synaptic activities is likely the consequence of adaptation, we mainly focused on the upstream genes involved in survival signaling. Six genes in focal adhesion pathway were significantly correlated with AD progression, including FN1, ITGB5, FLNA, PXN, FYN and CSK. In addition, FYN and CSK are Alzgene. Four genes in insulin signaling pathway were selected, among which IRS1, PIK3R1, SOS2 are Alzgene and dys-regulated during aging. FOXO1 were significantly correlated with AD progression and also dys-regulated during aging. Five genes in neurotrophin signaling were selected, among which SOS2, NFKBIA, GSK3B are Alzgenes. SOS2 is an important neurotrophin/insulin signal mediator. GSK3B regulates cytoskeleton and the formation of axon and synapse, and it is likely even more critical because it’s a major tau kinase. Among the five selected genes involved in apoptosis, TNFRSF1A, CASP8, NFKBIA were Alzgene and also dys-regulated during aging, and TNFRSF1A and NFKBIA were significantly correlated with AD progression. NFKBIA is of special interest because it’s involved in both TNF-receptor signaling and neurotrophin signaling. HATs (KAT2B, CREBBP and EP300) and HDAC (HDAC1) are related to learning and memory, and all four genes were significantly correlated with AD progression and also dys-regulated during aging. Steroid hormone receptors (nuclear receptor subfamily 3) NR3C1, AR and ESR1 protect neurons from beta-amyloid and AD-associated neurotoxicity, and all three genes are Alzgene and dys-regulated during aging. Additionally, PPP2CA can dephosphorylate tau and was significantly correlated with AD progression. Housekeeping gene GAPDH as an Alzgene was significantly correlated with AD progression and was also found in amyloid plaques and tangles. Finally, the importance of APP, MAPT and YWHAZ was also supported by multiple evidences.

### Comparison with Previous Studies on AD Co-expression Network

In a recent work, Liu et al. conducted a study to find active subnetworks using their novel scoring function based on combining co-expression information of the edges and the differential expression of the nodes in these six brain region [21]. They initiated the search for significant subnetwork from a pre-built AD related PPI network via prior AD knowledge. Here we attempted *de novo* discovery of significant subnetwork in the entire human protein interactome. In both studies, it was found that perturbed subnetworks in different brain regions were significantly overlapped with each other, suggesting that some important common features may be shared among different brain regions. However, Liu et al. mainly focused on their newly developed algorithm, and they started their search on a pre-built AD related network. In addition, they interpreted the significant subnetwork by comparing with known Alzheimer’s pathway in KEGG, which may cause the loss of information because unknown AD genes may also have significant effect on AD. On the other hand, our analysis is more comprehensive and less biased, and our hub network is proven to be very informative of AD pathogenesis (**Table S8**).

In another work, Ray et al. constructed an unweighted co-expression network by using gene expression data for the EC region to discover important hub genes [134]. They identified 107 hub genes, among which only NFKBIA and LNX1 gene are shared with the hub genes identified in this work. This low overlap is primarily due to the difference in the topological structures between co-expression network and PPI network and heterogeneity in transcriptome data. Due to the high noise nature of omics data, integrating multiple sources of information is generally preferred over relying on a single source of omics data, which is why we combined expression information with PPI information to identify critical genes. Apart from that, different analytical methods and criteria for the hub gene selection may also contribute to the low overlap. Compared to the work by Ray et al., our work provided more extensive evidence on the biological relevance of the identified hub genes (listed in **Table S8**). Nevertheless, when comparing functional enrichment results, the perturbed subnetworks found in both studies are associated with metabolism and biosynthesis, transcription, intracellular signal transduction, protein kinase and phosphorylation, cell organization, protein transport and neuron development.

In this work, we conducted comprehensive network analyses on the dys-regulation of gene expression in six brain regions of Alzheimer’s disease. We found perturbed subnetworks in these six brain region which were significantly overlapped with each other. Based on the perturbed subnetworks, we further identified 142 hub genes, 136 of which formed a connected network. The biological relevance of the hub network has been supported by multiple lines of evidence. Many of the genes in the hub network were components of plaques or tangles based on previous proteomics studies. In the meanwhile, many of the genes were linked to aging or genetic risk of AD. By examining the correlation of each hub gene’s expression with MMSE and NFT scores that quantifies AD progression, we found that this hub network may play a vital role in the disease progression. In addition, AD-specific perturbation of the hub network has been confirmed by comparing with other related diseases. Most importantly, we here propose that the hub network reflects the adaptation strategy of “healthy” neurons in AD specific micro-environment.

## Methods

### Data Processing for Gene Expression and Human Protein-protein Interaction

All microarray data were downloaded from Gene Expression Omnibus (GEO). In the major dataset GSE5281 [5], “healthy” neurons were collected by laser-capture microdissection from six different brain regions which are either histo-pathologically or metabolically relevant to AD. The study population consisted of 13 normal elderly controls (NED) and 10 AD patients for entorhinal cortex (EC), 13 NEDs and 10 AD patients for hippocampus (HIP), 12 NEDs and 16 AD patients for middle temporal gyrus (MTG), 13 NEDs and 9 AD patients for posterior cingulate cortex (PC), 12 NEDs and 19 AD patients for primary visual cortex (VCX), and 11 NEDs and 23 AD patients for superior frontal gyrus (SFG). Other major datasets included GSE15222, GSE1297, GSE6613, GSE11882 for late-stage AD cortex, disease progression of AD in HIP region, AD blood and aging, respectively. For protein interaction data, we utilized a dataset of literature-curated human protein-protein interactions (PPIs) from the Human Protein Reference Database (HPRD) [135], comprising 36504 interactions among 9386 genes at the time of download.

Preprocessing of microarray data was performed in R (http://cran.r-project.org/), a freely available platform, using microarray-specific packages available through Bioconductor (http://www.Bioconductor.org/). Raw data (CEL files) were processed with the “mas5” function in the “affy” library of Bioconductor to achieve global scaling with target intensity of “150” for all probe sets. Probe sets called in fewer than 10% of total arrays with the “mas5calls” function in the “affy” library were considered unreliable. Probe sets not reliably detected in at least three brain regions were removed from further analysis. Control probe sets and probe sets not associated with known genes were also removed from further analysis. If multiple probe sets represented the same gene, the probe set with the highest variance was used. The Affymetrix probe set IDs and HPRD gene symbols were mapped to Entrez Gene IDs. For HPRD, self-loops were removed, and proteins without expression value were also removed. The remaining largest connected component was kept as the PPI network for further analyses**.** Expression matrix was reduced to genes present in the PPI network and processed for differential expression by employing the two class Linear Models for Microarray Data (LIMMA) with the Limma package in R [28].

### Identification of Significantly Perturbed Subnetworks

A network search algorithm is required to find significantly perturbed subnetworks. We applied a heaviest induced subgraph algorithm (Heinz) that computes optimal and suboptimal solutions to the maximal-scoring subgraph (MSS) problem using integer linear programming [24]. Before searching for significantly perturbed subnetworks with maximal aggregated scores, each individual gene in the network was assigned to a differential significance score based on the raw P-values calculated from differential expression analysis. The distribution of the raw P-values can be considered as a mixture of signal and noise, where the signal component is assumed to be Beta(a,1) distributed, and the noise is B(1,1)  =  uniform (0,1) distributed (a beta-uniform mixture (BUM) model). Thus, the differential significance score is given as.



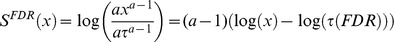
where x represents the raw P-values, a is the maximum-likelihood estimation of the shape parameter for the BUM model, which indicates the signal component is equal to the B(a,1) density, and τ is the significance threshold, which controls the FDR for the positively scoring P-values and fine-tunes the discrimination of signal and noise. P-values below the threshold (signal) will score positively whereas those above the threshold (noise) will be assigned negative scores. First, we fitted a beta-uniform mixture (BUM) model on the entire set of raw P-values of differential expression, from which the maximum-likelihood estimation of the fitted parameters for the BUM model can be obtained. For each brain region, we then scanned a set of FDRs and selected a FDR that assigns approximately 10% of the nodes on the PPI network as positively scoring signal component. The resulting FDR was 0.00009, 0.0004, 0.0008, 0.002, 0.01 and 0.05 for MTG, EC, HIP, PC, SFG and VCX region, respectively. Finally, based on the differential significance scores and the PPI network, significantly perturbed subnetworks were searched with the Heinz algorithm. An outline of the Heinz algorithm is as follows: First, all positive and connected nodes are aggregated into meta-nodes. Then, by defining an edge score based on the scores of nodes connected by the edge, the node scores are transferred to the edges. Based on the edge scores, a minimum spanning tree (MST) is then calculated. Lastly, all paths between positive meta-nodes are calculated based on the MST to obtain the negative nodes between the positives. Based on these negative nodes, again a MST is calculated from which the path with the highest score, regarding node scores of negative nodes and the positive meta-nodes connecting them, gives the final approximated subnetwork. All computational algorithms are implemented in the R BioNet package. For more detailed information on the algorithm and the software package please refer to the original paper [136].

### Identification of Hub Genes and Discovery of a Hub Network

For each brain region, the genes at the top of degree distribution (> = 90% quantile) in the significantly perturbed subnetworks were defined as hub genes. Those hub genes with pairwise interactions in at least one brain region’s perturbed subnetworks were connected to form a hub network.

### Functional Enrichment Analyses

To identify the biological functions of the significantly perturbed subnetworks, the genes within the perturbed subnetwork in each brain region were analyzed by various tools, including Database for Annotation, Visualization and Integrated Discovery (DAVID) for Gene Ontology (GO) enrichment [137], WEB-based Gene Set Analysis Toolkit (WebGestalt) for transcription factor binding sites (TFBS) enrichment [138], and kinase enrichment analysis (KEA) for kinase substrate enrichment [139]. In the enrichment analyses, more than 5 genes present and p<0.01 for a category are required to be considered significant. For the hub network, we also performed KEGG (Kyoto Encyclopedia of Genes and Genomes) pathway enrichment using DAVID, and drug target enrichment analysis using ToppFun [140], which integrates Stitch, CTD and Drug Bank databases. Enriched GO-categories were organized into a network by EnrichmentMap [141] for better visualization, where the edges were defined by the overlap coefficient between the GO categories (overlap coefficient cut-off 0.5).

### Perturbation of the Hub Network in AD and Other Related Diseases

We downloaded microarray datasets from GEO for other related diseases, including GSE20168, GSE20291, GSE20292 [142] and GSE7621 [143] for Parkinson’s disease (PD), GSE3790 [144] for Huntington’s disease (HD), and GSE12654 [145] and GSE17612 [146] for schizophrenia (SZ). All data were processed following the same procedure described above. In order to validate the robust perturbation of the hub network, we chose another AD dataset GSE15222 with the largest sample size so far (364 subjects in total) [147]. For this dataset, only series matrix file was available. Probe sets with negative or missing expression value in more than 10% of the total array were removed. For probe sets with negative or missing expression values in less than 10% the total array, missing values were imputed with the k-nearest neighbor algorithm (k-NN). 115 genes from the hub network were found after removal, including 19 genes with negative or missing values, which were filled in with the imputed values. Differential expression moderated t-statistics were calculated using LIMMA and the significance of hub network perturbation was determined by taking the average of the absolute moderated t-statistics of all genes within the hub network. Moderated t-statistics lead to P-values in the same way as ordinary t-statistics do except that the degrees of freedom are increased, reflecting the greater reliability associated with the smoothed standard errors.

### Correlation between the Hub Network and AD Progression

To investigate the correlation between the hub network and AD progression, we used microarray dataset GSE1297 [148], which studied hippocampal gene expression of subjects with varying AD severity. Subjects were initially classified into four groups, termed “Control” (MMSE >25), “Incipient AD” (MMSE 20–25), “Moderate AD” (MMSE 14–19), and “Severe AD” (MMSE <14). Microarray data (series matrix file) was processed following the same procedure according to the original paper. Pearson correlation analysis was performed for each gene of the hub network against both MMSE and NFT measures of each subject. When multiple probe sets correspond to the same gene, the probe set with the most significant correlation with MMSE or NFT scores was used. Genes were called significant if their expression values correlates with the MMSE, NFT, or both across all subjects at P-values < = 0.05 (significance threshold was set according to the original paper).

## Supporting Information

Figure S1 **A detailed flowchart for the analysis procedure.** The calculated differential expression p-values were fitted to a BUM model for noise reduction. Based on the fitted parameters, nodes in PPI network was scored and maximal scoring subnetwork was obtained by Heinz algorithm. From these perturbed subnetworks, the hub genes were extracted, which then formed a connected hub network. The biological relevance of the hub network was supported by additional analysis.
(PDF)

Figure S2 **An example of the BUM model fitting.** For EC region, the BUM model fits nicely with the empirical P-value distribution. **Left:** The histogram of the observed P-values (black color) shows good consistency with the expected densities under the fitted model (red line). The blue line indicates the fraction of P-values derived from the uniform noise model. **Right:** The good fitting of the model has also been confirmed by a Q–Q plot of the fitted distribution versus the observed P-value distribution.
(PDF)

Figure S3 **A Venn diagram showing the overlap of perturbed subnetworks in five brain regions (VCX region excluded due to technical difficulty in plotting).** The total number of nodes (edges) are 345 (514), 299 (422), 301 (447), 263 (332), 304 (447), and 283 (390) for MTG, EC, HIP, PC, SFG and VCX, respectively.
(PDF)

Figure S4 **An example of the perturbed subnetworks.** The subnetwork perturbed in HIP region is shown. Up-regulated genes are indicated by red color. Down-regulated genes are indicated by green color.
(PDF)

Table S1 **Enrichment of transcription factor targets in the perturbed subnetworks.** The analysis was performed by a web tool named WebGestalt. The search for conserved transcription factor binding sites and anonymous motifs was restricted to a sequence window corresponding to ±2 kb of the transcription start site. The subnetwork in each of the six brain regions was submitted to WebGestalt and the enrichment p-values of the binding motifs were returned. Only motifs with p-values <0.01 in at least 3 brain regions were selected.
(PDF)

Table S2 **Enrichment of kinase substrates in the perturbed subnetworks.** The analysis was performed by a web tool named KEA (kinase enrichment analysis). The subnetwork in each of the six brain regions was submitted to KEA and the enrichment p-values of the kinases were returned. Only kinases with p-values <0.01 in at least 3 brain regions were selected.
(PDF)

Table S3 **Perturbation of the hub network in AD and other related diseases including Parkinson’s disease (PD), Huntington’s disease (HD) and schizophrenia (SZ).** The numbers of genes with detected expression value in each microarray dataset are provided. The significance of perturbation was calculated by taking the average of the absolute t statistics of all genes in the hub network. A significance threshold of 0.05 was chosen in this work.
(PDF)

Table S4 **Genes in the hub network constituting amyloid plaques or neurofibrillary tangles according to previous proteomics studies.** Genes within both categories are indicated by **bold** font.
(PDF)

Table S5 **Genes in the hub network significantly correlated with AD progression according to MMSE sore or NFT score.** P< = 0.05 is considered significant. Genes correlated with both MMSE and NFT scores are shown in **bold**.
(PDF)

Table S6 **Genes in the hub network associated with genetic risk (ALZgene database) and aging (GenAge database).** Genes within both categories are indicated by **bold** font.
(PDF)

Table S7 **A**) Dys-regulation of the hub genes at three stages, including aging, intermediate stage with “healthy” neurons in AD specific environment, and late stage AD. Gene dys-regulation is presented by–log(p-value). Up-regulation is indicated by positive values and down-regulation is indicated by negative values. Significantly dys-regulated genes (p<0.01) are marked as red for up-regulation or green for down-regulation. **B**) Dys-regulation of genes in two microarray studies focused on the comparison NFT-bearing and NFT-free neurons. 63 genes in the perturbed subnetworks of the six brain regions were found dys-regulated in the Kramer 2008 study, including 7 hub genes as indicated by color-filled cells (red for up-regulation and green for down-regulation), The corresponding dys-regulation of these 63 genes in the six brain regions is provided as a reference. In another study (GSE4757), only a small number of dys-regulated genes were found, and the two dys-regulated hub genes are listed. **C**) Critical hub genes involved in survival signaling. The evidence for supporting the selection is provided, including the constituents of amyloid or tangle, correlation with AD progression based on MMSE or NFT score, genetic risk (ALZgene) and aging-related genes. Genes marked as red are discussed in the main text.
(XLSX)

Table S8 **A detailed comparison between this work and a previous work by Liu et al. on the network analysis of AD transcriptome.**
(PDF)
