# Supplementary figures and images for: Concerted Perturbation Observed in a Hub Network in Alzheimer’s Disease
Source: PLoS One. 2012 Jul 16;7(7):e40498. doi: 10.1371/journal.pone.0040498 (PMC3398025; doi:10.1371/journal.pone.0040498)

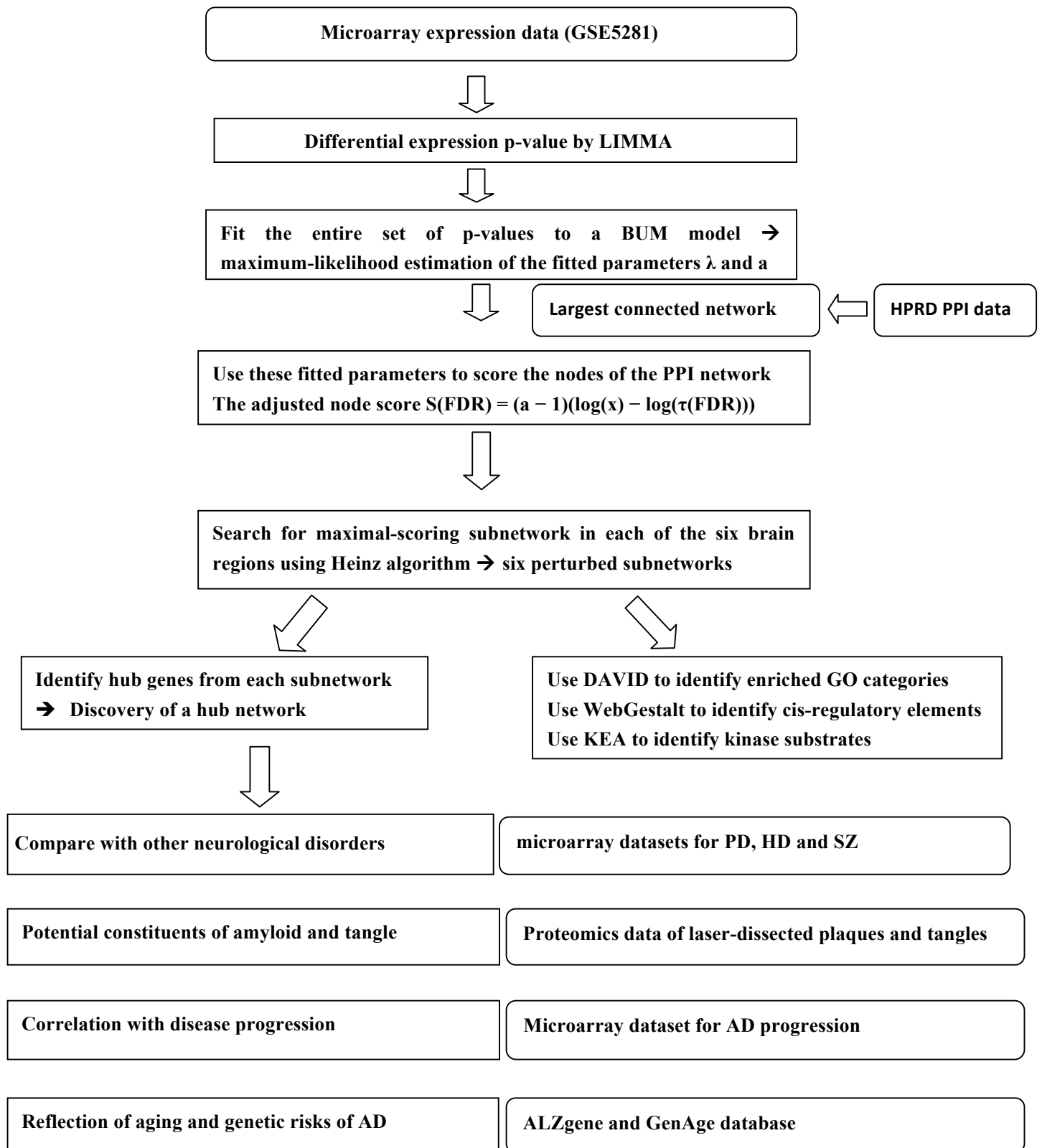

**Figure S1**

Supplement: Figure S1 — A detailed flowchart for the analysis procedure. The calculated differential expression p-values were fitted to a BUM model for noise reduction. Based on the fitted parameters, nodes in PPI network was scored and maximal scoring subnetwork was obtained by Heinz algorithm. From these perturbed subnetworks, the hub genes were extracted, which then formed a connected hub network. The biological relevance of the hub network was supported by additional analysis. (PDF) [file pone.0040498.s001.pdf]

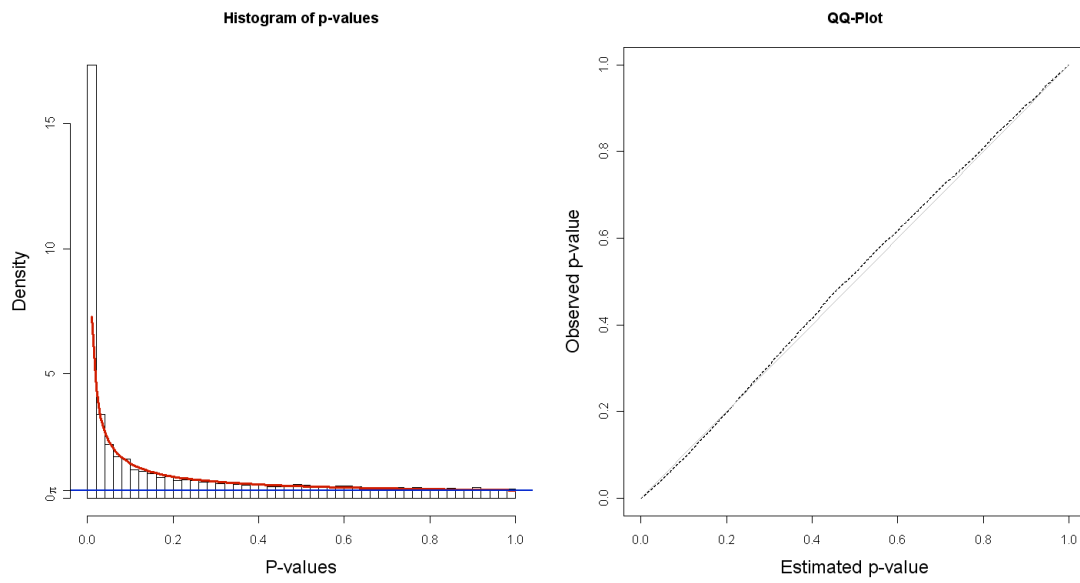

**Figure S2.**

Supplement: Figure S2 — An example of the BUM model fitting. For EC region, the BUM model fits nicely with the empirical P-value distribution. Left: The histogram of the observed P-values (black color) shows good consistency with the expected densities under the fitted model (red line). The blue line indicates the fraction of P-values derived from the uniform noise model. Right: The good fitting of the model has also been confirmed by a Q–Q plot of the fitted distribution versus the observed P-value distribution. (PDF) [file pone.0040498.s002.pdf]

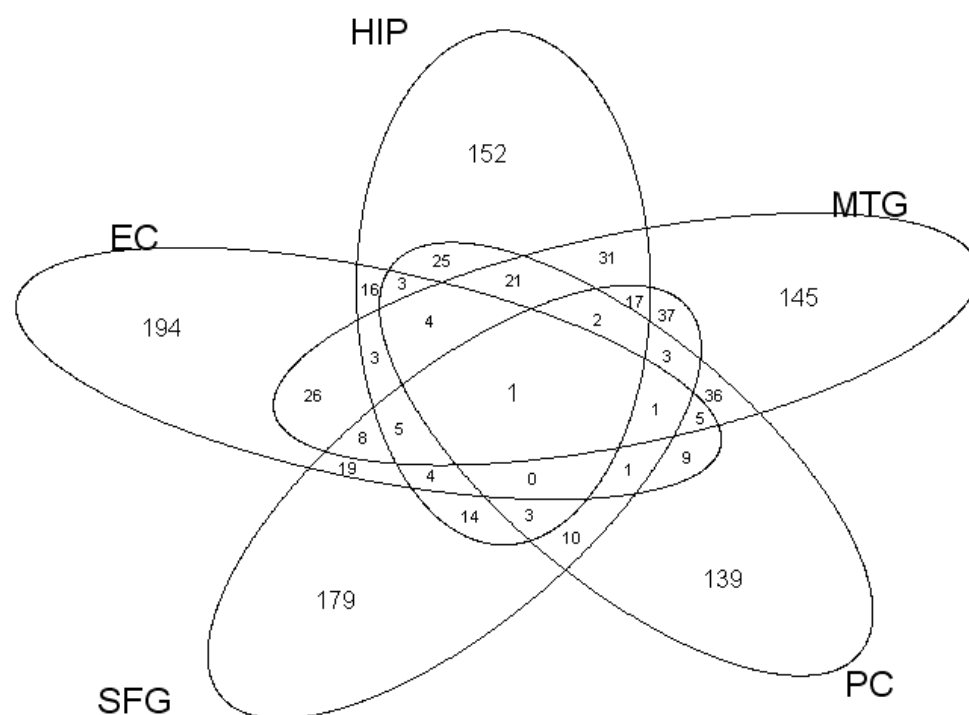

**Figure S3.**

Supplement: Figure S3 — A Venn diagram showing the overlap of perturbed subnetworks in five brain regions (VCX region excluded due to technical difficulty in plotting). The total number of nodes (edges) are 345 (514), 299 (422), 301 (447), 263 (332), 304 (447), and 283 (390) for MTG, EC, HIP, PC, SFG and VCX, respectively. (PDF) [file pone.0040498.s003.pdf]

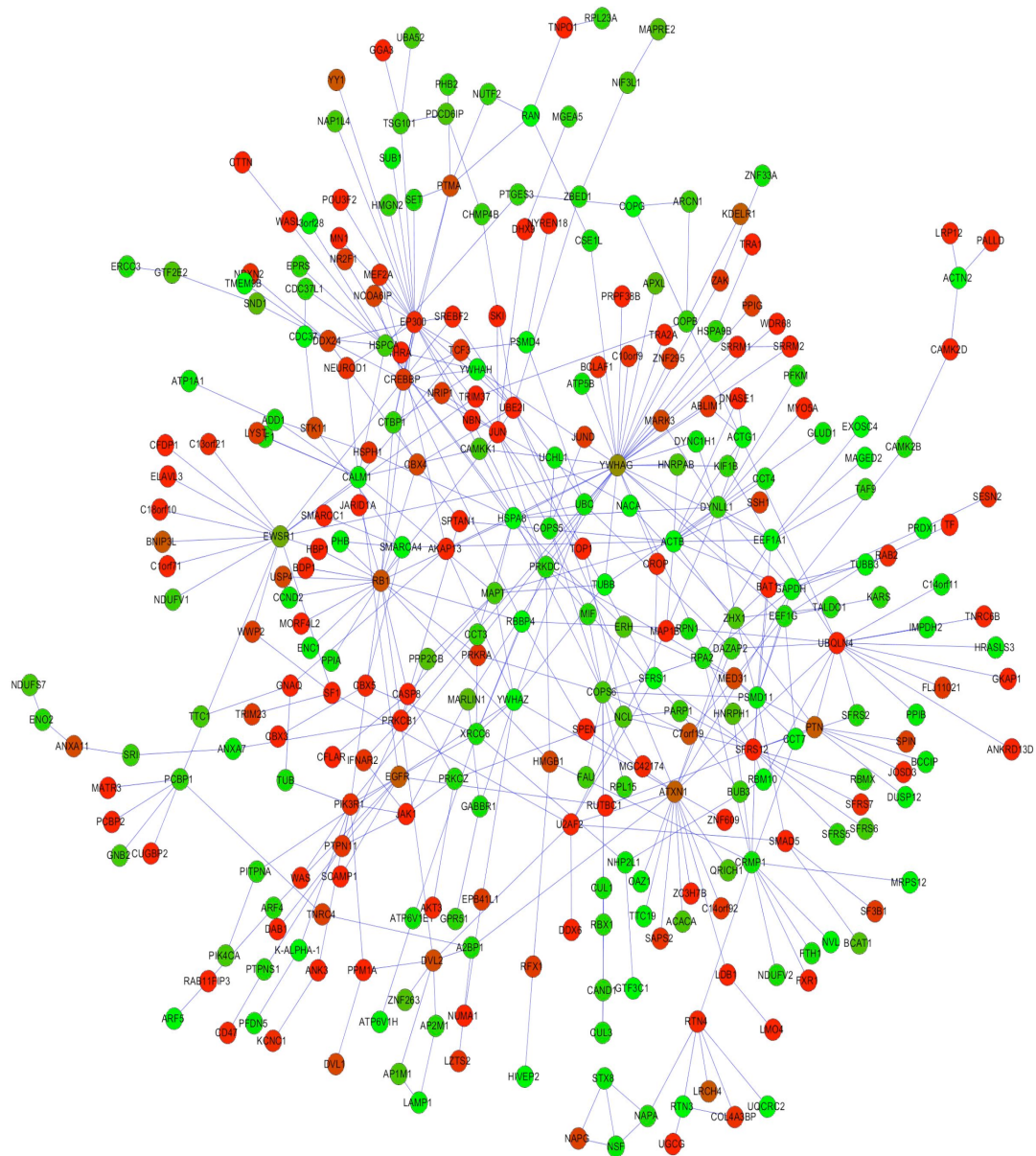

**Figure S4.**

Supplement: Figure S4 — An example of the perturbed subnetworks. The subnetwork perturbed in HIP region is shown. Up-regulated genes are indicated by red color. Down-regulated genes are indicated by green color. (PDF) [file pone.0040498.s004.pdf]
