# Supplementary material for: Concerted Perturbation Observed in a Hub Network in Alzheimer’s Disease
Source: PLoS One. 2012 Jul 16;7(7):e40498. doi: 10.1371/journal.pone.0040498 (PMC3398025; doi:10.1371/journal.pone.0040498)
Supplement: Table S1 — Enrichment of transcription factor targets in the perturbed subnetworks. The analysis was performed by a web tool named WebGestalt. The search for conserved transcription factor binding sites and anonymous motifs was restricted to a sequence window corresponding to ±2 kb of the transcription start site. The subnetwork in each of the six brain regions was submitted to WebGestalt and the enrichment p-values of the binding motifs were returned. Only motifs with p-values <0.01 in at least 3 brain regions were selected. (PDF) [file pone.0040498.s005.pdf]

Table S1.

**Transcription factor binding motif**	**Description**	**Number of regions enriched**	**Brain regions enriched**
CTTTGT_V$**LEF1**_Q2	Genes with promoter regions containing the motif CTTTGT which matches annotation for LEF1: lymphoid enhancer-binding factor 1	5	EC/HIP/MTG/SFG/VCX
GGGCGGR_V$**SP1**_Q6	Genes with promoter regions containing the motif NGGGGGCGGGGYN which matches annotation for SP1:Sp1 transcription factor	5	HIP/MTG/SFG/PC/VCX
GGGAGGRR_V$**MAZ**_Q6	Genes with promoter regions containing the motif GGGAGGRR which matches annotation for MAZ: MYC-associated zinc finger protein	4	HIP/MTG/SFG/VCX
GGGTGGRR_V$**PAX4**_03	Genes with promoter regions containing the motif GGGTGGRR which matches annotation for PAX4:paired box gene 4	4	EC/MTG/SFG/VCX
RCGCANGCGY_V$**NRF1**_Q6	Genes with promoter regions containing the motif TGACCTY which matches annotation for NRF1: nuclear respiratory factor 1	4	EC/HIP/SFG/VCX
TAATTA_V$**CHX10**_01	Genes with promoter regions containing the motif TAATTA which matches annotation for VSX1: visual system homeobox 1 homolog, CHX10-like (zebrafish)	4	EC/MTG/SFG/VCX
V$**SRY**_02	Genes with promoter regions containing the motif NWWAACAAWANN which matches annotation for SRY:sex determining region Y	4	HIP/MTG/SFG/VCX
CAGGTG_V$**E12**_Q6	Genes with promoter regions containing the motif CAGGTG which matches annotation for TCF3: transcription factor 3 (E2A immunoglobulin enhancer binding factors E12/E47)	3	EC/MTG/SFG
CATTGTYY_V$**SOX9**_B1	Genes with promoter regions containing the motif CATTGTYY which matches annotation for SOX9: SRY (sex determining region Y)-box 9 (campomelic dysplasia, autosomal sex-reversal)	3	HIP/MTG/SFG
GCCATNTTG_V$**YY1**_Q6	Genes with promoter regions containing the motif GGGTGGRR which matches annotation for YY1:YY1 transcription factor	3	HIP/MTG/VCX
TGACCTY_V$**ERR1**_Q2	Genes with promoter regions containing the motif TGACCTV which matches annotation for ESRRA:estrogen-related receptor alpha	3	EC/MTG/SFG
TTGTTT_V$**FOXO4**_01	Genes with promoter regions containing the motif TTGTTT which matches annotation for MLLT7: myeloid/lymphoid or mixed-lineage leukemia (trithorax homolog, Drosophila); translocated to, 7	3	EC/MTG/SFG

V$**ARNT**_02	Genes with promoter regions containing the motif NNNNNRTCACGTGAYNNNNN which matches annotation for ARNT: aryl hydrocarbon receptor nuclear translocator	3	HIP/MTG/PC
V$**CREB**_Q4_01	Genes with promoter regions containing the motif CNNTGACGTMA which matches annotation for CREB1: cAMP responsive element binding protein 1	3	MTG/PC/VCX
V$**E2F**_Q6_01	Genes with promoter regions containing the motif NKCGCGCSAAAN which matches annotation for E2F TFDP1: transcription factor Dp-1	3	HIP/MTG/VCX
V$**HLF**_01	Genes with promoter regions containing the motif GTTACRYAAT which matches annotation for HLF: hepatic leukemia factor	3	HIP/MTG/SFG
YGCANTGCR_UNKNOWN	Genes with promoter regions containing the motif YGCANTGCR. Motif does not match any known transcription factor	3	MTG/PC/VCX
CTGCAGY_UNKNOWN	Genes with promoter regions containing the motif CTGCAGY. Motif does not match any known transcription factor	3	EC/MTG/VCX
SMTTTTGT_UNKNOWN	Genes with promoter regions containing the motif SMTTTTGT. Motif does not match any known transcription factor	3	EC/HIP/MTG
