# Supplementary material for: Concerted Perturbation Observed in a Hub Network in Alzheimer’s Disease
Source: PLoS One. 2012 Jul 16;7(7):e40498. doi: 10.1371/journal.pone.0040498 (PMC3398025; doi:10.1371/journal.pone.0040498)
Supplement: Table S2 — Enrichment of kinase substrates in the perturbed subnetworks. The analysis was performed by a web tool named KEA (kinase enrichment analysis). The subnetwork in each of the six brain regions was submitted to KEA and the enrichment p-values of the kinases were returned. Only kinases with p-values <0.01 in at least 3 brain regions were selected. (PDF) [file pone.0040498.s006.pdf]

Table S2.

Kinase	Number of brain regions enriched	Brain regions enriched
IKBKE	5	EC/HIP/MTG/PC/VCX
BCR	4	EC/HIP/MTG/VCX
CHUK	4	HIP/MTG/PC/VCX
IKBKB	4	HIP/MTG/PC/VCX
PRKCD	4	HIP/MTG/SFG/VCX
AKT1	3	EC/HIP/VCX
CDK5	3	EC/MTG/PC
CSK	3	EC/MTG/SFG
MAP3K3	3	HIP/PC/VCX
MAP3K7	3	HIP/MTG/VCX
MAPK3	3	MTG/PC/VCX
MAPK8	3	MTG/PC/VCX
MARK4	3	HIP/MTG/PC
PRKCZ	3	MTG/PC/VCX
RAF1	3	HIP/MTG/PC
RIPK1	3	HIP/MTG/PC
RIPK2	3	HIP/MTG/PC
RIPK3	3	HIP/MTG/PC
