# Supplementary material for: Concerted Perturbation Observed in a Hub Network in Alzheimer’s Disease
Source: PLoS One. 2012 Jul 16;7(7):e40498. doi: 10.1371/journal.pone.0040498 (PMC3398025; doi:10.1371/journal.pone.0040498)
Supplement: Table S3 — Perturbation of the hub network in AD and other related diseases including Parkinson’s disease (PD), Huntington’s disease (HD) and schizophrenia (SZ). The numbers of genes with detected expression value in each microarray dataset are provided. The significance of perturbation was calculated by taking the average of the absolute t statistics of all genes in the hub network. A significance threshold of 0.05 was chosen in this work. (PDF) [file pone.0040498.s007.pdf]

Table S3.

**Dataset_Disease_Region**	**Number of genes detected**	**P-value**
GSE12654_SZ_prefontal _BA10	110	0.382
GSE17612_SZ_prefontal_BA10	129	0.423
GSE20168_PD_prefontal_BA9	123	0.0837
GSE20291_PD_putamen	122	0.356
GSE20292_PD_substantia nigra	120	0.214
GSE7621_PD_substantia nigra	134	0.253
GSE3790_HD_prefontal_BA4	126	0.0725
GSE3790_HD_prefontal_BA9	127	0.561
GSE3790_HD_cerebellum	125	0.206
GSE5281_AD_EC	130	**0.0191**
GSE5281_AD_HIP	133	**0.00547**
GSE5281_AD_MTG	134	**0.00111**
GSE5281_AD_PC	134	**0.0121**
GSE5281_AD_SFG	135	**0.0312**
GSE5281_AD_VCX	134	0.0606
GSE15222_AD_cortex (frontal, temporal, parietal, cerebellar)	115	**2.58E-05**
