# Supplementary material for: Concerted Perturbation Observed in a Hub Network in Alzheimer’s Disease
Source: PLoS One. 2012 Jul 16;7(7):e40498. doi: 10.1371/journal.pone.0040498 (PMC3398025; doi:10.1371/journal.pone.0040498)
Supplement: Table S4 — Genes in the hub network constituting amyloid plaques or neurofibrillary tangles according to previous proteomics studies. Genes within both categories are indicated by bold font. (PDF) [file pone.0040498.s008.pdf]

Table S4.

plaque	tangle
**SNCA**	**SNCA**
**ACTB**	**ACTB**
**MAPK1**	**MAPK1**
**MAPT**	**MAPT**
**HSPA8**	**HSPA8**
**HNRNPK**	**HNRNPK**
**GAPDH**	**GAPDH**
**EEF1A1**	**EEF1A1**
**DNM1**	**DNM1**
**CLTC**	**CLTC**
**TUBA4A**	**TUBA4A**
CDC42	TUBB
DYNLL1	UBC
YWHAZ	
YWHAH	
YWHAG	
YWHAB	
SNAP25	
RTN4	
MAP2K1	
PRKCA	
PEBP1	
APP	
HSP90AA1	
HSPA1A	
XRCC6	
FLNA	
DLG4	
YWHAQ	
CRMP1	
PCBP1	
