# Supplementary material for: Concerted Perturbation Observed in a Hub Network in Alzheimer’s Disease
Source: PLoS One. 2012 Jul 16;7(7):e40498. doi: 10.1371/journal.pone.0040498 (PMC3398025; doi:10.1371/journal.pone.0040498)
Supplement: Table S5 — Genes in the hub network significantly correlated with AD progression according to MMSE sore or NFT score. P< = 0.05 is considered significant. Genes correlated with both MMSE and NFT scores are shown in bold. (PDF) [file pone.0040498.s009.pdf]

**Table S5**.

| *Gene symbol* | *MMSE_cor* | *Gene symbol* | *NFT_cor* |
|---|---|---|---|
| **FOXO1** | 0 | **KPNB1** | 0 |
| **ITGB5** | 0 | **FOXO1** | 8.00E-04 |
| **CLTC** | 5.00E-04 | **ITGB5** | 0.0013 |
| **YWHAZ** | 9.00E-04 | **CSK** | 0.0014 |
| **EP300** | -9.00E-04 | EEF1A1 | 0.0016 |
| **NFKBIA** | -0.0011 | **FLNA** | 0.0016 |
| **KPNB1** | -0.0012 | **CCNH** | -0.0017 |
| **CRMP1** | 0.0014 | **CREBBP** | 0.002 |
| **EWSR1** | -0.0015 | **MAPK1** | 0.0028 |
| **FLNA** | -0.0016 | COPS5 | -0.0029 |
| **PPP2CA** | 0.0017 | **CRMP1** | -0.0033 |
| **PRKCB** | 0.002 | RTN4 | -0.0035 |
| **APP** | -0.0024 | **CLTC** | -0.0037 |
| **CSNK2A1** | 0.0028 | **TDG** | 0.0043 |
| **CDC42** | 0.0029 | **HSPA8** | -0.0044 |
| **TUBB** | 0.0031 | **DYNLL1** | -0.0047 |
| **PARD3** | -0.0031 | **GAPDH** | -0.0047 |
| **RPS6KA5** | -0.0032 | **YWHAZ** | -0.0053 |
| EGFR | -0.0047 | **NFKBIA** | 0.0057 |
| **DYNLL1** | 0.0048 | **APP** | 0.0065 |
| **YWHAH** | 0.0059 | UBC | -0.0071 |
| SUMO1 | 0.0082 | TSG101 | -0.0079 |
| **MAPK1** | -0.0084 | **SMAD5** | 0.0081 |
| **PTN** | 0.0092 | **EP300** | 0.0097 |
| SMAD4 | -0.0096 | BRCA1 | 0.0108 |
| **YWHAB** | 0.0099 | **SNAP25** | -0.0109 |
| **CSK** | -0.01 | **PTN** | -0.0116 |
| **SNAP25** | 0.0105 | **PARD3** | 0.0117 |
| **CREBBP** | -0.0115 | **JUN** | 0.0123 |
| ATXN1 | 0.0133 | **FN1** | -0.0149 |
| **TDG** | -0.0134 | PSMD11 | -0.0151 |
| **GAPDH** | 0.0148 | **PRKCB** | -0.0164 |
| **CDK5** | 0.015 | **FYN** | 0.0168 |
| **TNFRSF1A** | -0.0163 | **YWHAH** | -0.0183 |
| **HDAC1** | -0.0168 | **EWSR1** | 0.0184 |
| **APEX1** | 0.0185 | EEF1G | 0.0189 |
| HSP90AA1 | -0.0187 | **APEX1** | -0.0207 |

| | | | |
|---|---|---|---|
| **MAP3K14** | -0.0191 | **TNFRSF1A** | 0.0207 |
| CDK2 | -0.0196 | PDGFRB | 0.0221 |
| **CCNH** | 0.0205 | **CSNK2A1** | -0.0231 |
| **HNRNPK** | 0.0232 | **PXN** | 0.0233 |
| SNCA | 0.0248 | STAT1 | -0.0238 |
| SMAD1 | -0.0262 | **EIF5** | -0.0247 |
| **FN1** | 0.0274 | PEBP1 | 0.025 |
| **HSPA8** | 0.0283 | **TUBB** | -0.0254 |
| **FYN** | -0.0301 | **HNRNPK** | -0.0275 |
| U2AF2 | -0.0317 | GSK3B | 0.0279 |
| CALM1 | 0.0328 | **CDK5** | -0.0323 |
| **SMAD5** | -0.0343 | **PPP2CA** | -0.0338 |
| KAT2B | -0.0359 | **MAP3K14** | 0.034 |
| **PXN** | -0.0361 | PCNA | -0.0342 |
| SHC1 | -0.0371 | **YWHAB** | -0.036 |
| **PTMA** | -0.0372 | **HDAC1** | 0.0383 |
| **EIF5** | 0.0418 | **RPS6KA5** | 0.0388 |
| MAP2K1 | 0.0425 | **CDC42** | -0.0403 |
| **JUN** | -0.0462 | CTBP1 | 0.0411 |
| GSK3B | -0.0703 | **PTMA** | 0.0417 |
| PDGFRB | -0.0721 | MCM7 | 0.0461 |
| BRCA1 | -0.0747 | RGS2 | -0.0462 |
| RTN4 | 0.0758 | SMAD1 | 0.0589 |
| EEF1A1 | -0.0986 | SUMO1 | -0.0602 |
| COPS5 | 0.1119 | MAP2K1 | -0.0653 |
| PEBP1 | -0.1139 | CALM1 | -0.0799 |
| TSG101 | 0.1476 | ATXN1 | -0.0856 |
| UBC | 0.154 | SNCA | -0.1254 |
| STAT1 | 0.1728 | HSP90AA1 | 0.1638 |
| PSMD11 | 0.236 | U2AF2 | 0.1736 |
| MCM7 | -0.2606 | SHC1 | 0.2562 |
| CTBP1 | -0.302 | EGFR | 0.2659 |
| PCNA | 0.3086 | KAT2B | 0.6531 |
| RGS2 | 0.3204 | CDK2 | 0.7921 |
| EEF1G | -0.5812 | SMAD4 | -0.7954 |
