# Supplementary material for: Concerted Perturbation Observed in a Hub Network in Alzheimer’s Disease
Source: PLoS One. 2012 Jul 16;7(7):e40498. doi: 10.1371/journal.pone.0040498 (PMC3398025; doi:10.1371/journal.pone.0040498)
Supplement: Table S6 — Genes in the hub network associated with genetic risk (ALZgene database) and aging (GenAge database). Genes within both categories are indicated by bold font. (PDF) [file pone.0040498.s010.pdf]

Table S6.

ALZ	*AGING*
**YWHAZ**	**YWHAZ**
**UBE2I**	**UBE2I**
**PIK3R1**	**PIK3R1**
**NFKBIA**	**NFKBIA**
**MAPT**	**MAPT**
**LRP2**	**LRP2**
**AR**	**AR**
**IRS1**	**IRS1**
**APP**	**APP**
**HSPA1A**	**HSPA1A**
**GSK3B**	**GSK3B**
**NR3C1**	**NR3C1**
**ESR1**	**ESR1**
**APEX1**	**APEX1**
BAG3	CDC42
CASP8	SUMO1
SOS2	STAT3
SNCA	BRCA1
ATXN1	SHC1
PLCG1	RB1
SMAD3	MAPK3
GAPDH	PRKDC
FYN	PRKCA
TUBB	PDGFRB
CSK	PCNA
CDK5	JUN
TNFRSF1A	HSP90AA1
	HSPA8
	HMGB1
	HDAC1
	HTT
	XRCC6
	FOXO1
	AKT1
	EP300
	EGFR
	EEF1A1

	CREBBP
	CEBPB
	PTPN11
