# Supplementary material for: Concerted Perturbation Observed in a Hub Network in Alzheimer’s Disease
Source: PLoS One. 2012 Jul 16;7(7):e40498. doi: 10.1371/journal.pone.0040498 (PMC3398025; doi:10.1371/journal.pone.0040498)
Supplement: Table S8 — A detailed comparison between this work and a previous work by Liu et al. on the network analysis of AD transcriptome. (PDF) [file pone.0040498.s012.pdf]

Table S8.

	This work	***Previous work by Liu et. al.***
**Perturbed subnetwork**	*De novo* discovery from human interactome	Discovery from pre-built network for AD
**Algorithm**	Noise reduction (BUM model) and exact solution of MSS	Differential expression and co-expression
**Evidence of biological relevance**	Disease progression<br>Genetic risk and aging<br>Amyloid/tangle constituents<br>Robust and specific	Not provided
**Specific state of neuron**	aging<br>"healthy" neurons in AD<br>late-stage AD	Non-discriminative pathological condition
**Data mining**	Efforts on the manual integration and synthesis of literatures and knowledge	Focused on the explanation of active pathways underlying KEGG AD genes
**Disease mechanism**	adaptation strategy<br>formation of amyloid and NFT	formation of amyloid and NFT.
